# Methods for Improving the Germination of *Rhodotypos scandens* (Thunb.) Makino Seeds through Endocarp Removal

**DOI:** 10.3390/plants13050648

**Published:** 2024-02-26

**Authors:** Hyeon Min Kim, Jun Hyeok Kim, Mi Hyun Lee, Gun Mo Kim, Chung Youl Park, Da Hyun Lee, Chae Sun Na

**Affiliations:** 1 Division of Wild Plant and Seeds, Baekdudaegan National Arboretum, Bonghwa 36209, Republic of Korea; khm0766@koagi.or.kr (H.M.K.); kyocera9@hanmail.net (C.Y.P.);; 2 Experiment & Analysis Division, Incheon International Airport Regional Office, Animal and Plant Quarantine Agency, Incheon 22382, Republic of Korea

**Keywords:** cold stratification, endocarp removal, fluridone, *Rhodotypos scandens* (Thunb.) Makino, seed propagation, water absorption

## Abstract

*Rhodotypos scandens* (Thunb.) Makino is known to have a seed dispersal that is thick and stony (endocarp + seeds) and has potential as a landscaping tree seed. In several Rosaceae species, seeds are covered with a hard endocarp, making the internal seeds water-impermeable and germination difficult. Here, we analyzed the morphoanatomical traits and germination properties of *R*. *scandens* seeds. To identify ideal seed propagation conditions, we immersed *R*. *scandens* seeds in sulfuric acid for varying durations and subjected them to phytohormone (gibberellic acid A3 and fluridone) and a cold stratification (CS) (5 °C) treatment after endocarp removal (ER). The *R*. *scandens* stony seeds did not increase in mass by ≥25.0%. Following ER, the seed mass increased by ≥50.0% with water absorption when compared to the initial dry mass. Seed surfaces showed damage and cracks through scarification after 1 h of immersion in sulfuric acid, failing to germinate. A combination of ER, phytohormone treatment, and CS improved seed germination compared to ER alone (26.0 ± 5.3%). Overall, *R*. *scandens* seeds showed a dispersal with a hard endocarp from the parent plant, and a pre-treatment with ER, phytohormones, and CS was required for effective seed propagation.

## 1. Introduction

Global warming has led to rapid alterations in plant habitats and ecosystems. The loss of genetic diversity is one of the most critical consequences of these changes [1]. Seeds play a crucial role in the conservation of plant genetic resources both in situ and ex situ, making seed preservation and the acquisition of fundamental information indispensable for the preservation of genetic diversity [2]. Seed banks serve as repositories for storing seeds, which can be utilized for research and cultivation. Traditionally, seed banks have been conducive for the conservation of crop species. In recent decades, there has been a growing trend among botanical gardens and various plant institutions to establish seed banks dedicated to the conservation of wild plant species [3,4]. The Society for Ecological Restoration (SER) manages a Seed Information Database (SID) that accumulates seed-related data, including information on the weight, storage behavior, germination requirements, and other traits of more than 50,000 plant species [5]. The Baekdudaegan National Arboretum possesses and manages both the Seed Vault and Seed Bank for ex situ conservation against plant extinction, particularly that of wild plant species, highlighting Korea’s significant interest in seed-based conservation. However, collecting and storing wild plant seeds in a seed bank for ex situ conservation does not conclusively solve conservation issues. In order for the stored seeds to be used for future ecological restoration, they must meet the conditions that allow germination. Many studies on seeds are actively collecting information related to the germination requirements and environmental conditions necessary for plant reproduction. However, there remains a need for extensive research to overcome seed dormancy and determine the optimal germination conditions for numerous plant species. Understanding seed dormancy and germination is equally crucial for the collection and storage of diverse plant species.

Seed germination is a critical factor in the establishment and propagation of plant seedlings in their natural environment. In addition, the successful germination of seeds from an endangered species not only aids in the maintenance of regeneration but also plays an essential role in the ex situ conservation of the species [6,7]. Various factors, such as temperature, moisture, humidity, and light, play crucial roles in seed germination, with seed dormancy being the most significant determinant [8]. Seed dormancy is an evolutionary adaptation that inhibits germination under unfavorable environmental conditions [9,10]. In temperate regions, this dormancy strategy confers advantages as it prevents the seedling’s emergence before winter, which would otherwise allow its survival under severe conditions [11]. Nevertheless, while dormancy serves as a valuable biological mechanism and holds significance in natural settings, it is often considered an unfavorable trait for cultivated crops, landscaping trees, and ornamental plants. Breaking dormancy in these species typically requires varying lengths of time, ranging from a few weeks to well over a year. Hence, understanding the mechanisms that underpin dormancy breakage and germination induction is crucial for agricultural and horticultural propagation [12,13,14].

Seed dormancy is categorized according to the developmental stage of the embryo, hard seed coat, water absorption capacity, inactivation of enzymes such as α-amylase, and the interactions between internal phytohormones, such as gibberellin acid (GA), cytokinin, and abscisic acid (ABA), in seeds [8,15]. Globally, 50–90% of wild plant species yield mature seeds that exhibit various types and levels of dormancy [16]. Seed dormancy is broadly classified into five types: physiological dormancy (PD), morphological dormancy (MD), morphophysiological dormancy (MPD), physical dormancy (PY), and combinational dormancy (PY + PD) [16,17,18,19]. Within the family Rosaceae, nondormancy and the occurrence of PD are commonly observed [9,16]. Various methods have been applied to overcome PD, including seed stratification [20], the use of regulatory phytohormones [21,22], and chemical solutions [23,24]. Nevertheless, within the Rosaceae family, particularly in the genera *Rubus* and *Prunus*, instances exist where a hard seed coat functions as a protective layer for the internal seed structure. In these species, the hard stony layer (endocarp + seeds) permits water permeability into the seed interior, but water absorption occurs at a slow rate, thereby inhibiting germination until the seeds undergo a dormancy release treatment [25,26]. The hard stony layer is commonly perceived to exhibit dormancy in order to resist germination, yet endocarp removal may have the potential to promote or enhance germination. In general, once the dormancy enforced by the seed coat is alleviated, seed germination can initiate [27]. In addition, endocarp removal reduces the cold (0–10 °C) and/or warm (≥15 °C) stratification time required for dormancy release and enhances the germination of non-stratified seeds [28,29].

*Rhodotypos scandens* (Thunb.) Makino (English name: black jetbead), the only species in the *Rhodotypos* genus, is a deciduous shrub belonging to the Rosaceae family. This species is native to North-Central and Southeastern China, Japan, and Korea, growing to 2–3 m tall [30,31,32]. As the *R*. *scandens* tree grows well in dry soil and receives a lot of sunlight, it is a landscaping tree planted in gardens, roadsides, and parks. This species exhibits excellent growth in warm climates, and its flowers, which bloom in May, are colorful but continue to blossom throughout the season in warm regions. Therefore, it belongs to the landscape tree category with the potential for high ornamental value.

The aim of this study was to investigate the effect of thick endocarp removal (ER) on the germination of *R*. *scandens* seeds in Korea and to introduce this as a novel germination method. Second, we conducted a basic analysis of the external and internal morphoanatomical characteristics of the seeds. Finally, we investigated the germination characteristics of the seeds following ER and various combination treatments involving phytohormones (gibberellic acid and fluridone) and cold stratification (CS). The current findings offer fundamental insights that could potentially be utilized for restoration or propagation techniques, particularly with regard to germination characteristics in order to achieve the stable production of *R*. *scandens* seeds.

## 2. Results

### 2.1. Seed Characteristics and Water Imbibition Test

**Figure 1:**
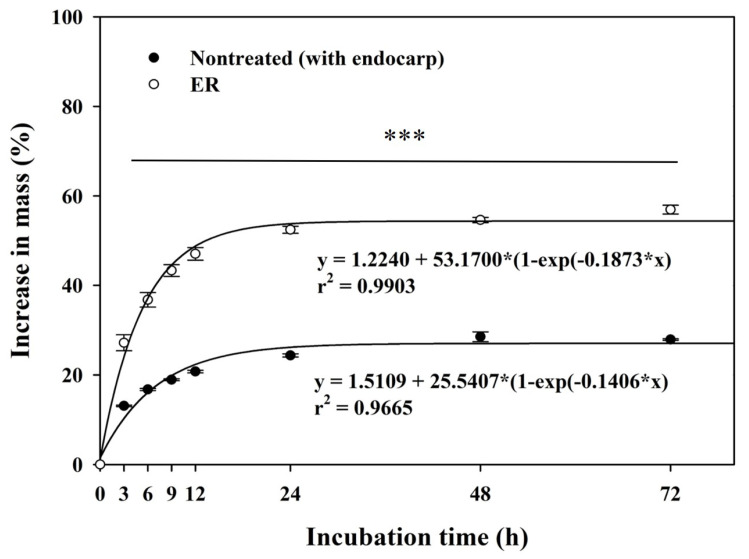
Changes in the mass of the *Rhodotypos scandens* (Thunb.) Makino seeds with and without endocarp during water incubation. The seeds were incubated at room temperature (approximately 23 ± 2 °C) on filter paper moistened with distilled water for 72 h. Vertical bars represent the standard deviation (*n* = 4). A paired *t*-test was employed to compare water absorption at different time points (3, 6, 9, 12, 24, 48, and 72 h) between those nontreated and those without endocarp. *** Significant at *p* ≤ 0.001. ER, endocarp removal.

The sampled *R*. *scandens* seeds displayed a spherical shape, measuring 8.394 ± 0.078 mm in length and 5.557 ± 0.074 mm in width, with an average mass of 130.420 ± 0.994 g per 1000 seeds. The water imbibition experiment conducted to assess seed permeability revealed that, after 3 h, the ER seeds had absorbed significantly more water compared to nontreated seeds (with endocarp) (Figure 1). The mass of the ER seeds increased by 27.18 ± 1.78% within 3 h and by 52.45 ± 0.79% within 24 h. In contrast, the mass of nontreated (with endocarp) seeds exhibited a mere 13.10 ± 0.17% increase within 3 h and a 24.36 ± 0.36% increase within 24 h. The mass of nontreated (with endocarp) seeds exhibited a 28.52 ± 1.09% increase after 48 h, which was maintained consistently throughout the experiment. After 72 h of water imbibition, the weight of the ER seeds increased by 28.22–29.86% more than that of the nontreated (with endocarp) seeds. The water absorption rate between the nontreated (with endocarp) seeds and the ER seeds exhibited a significant difference (*p* ˂ 0.05). The rigid and compact seed endocarp is the primary impediment to imbibition in *R*. *scandens* seeds.

### 2.2. Structural Analysis of Internal and External Seed Morphoanatomy

**Figure 2:**
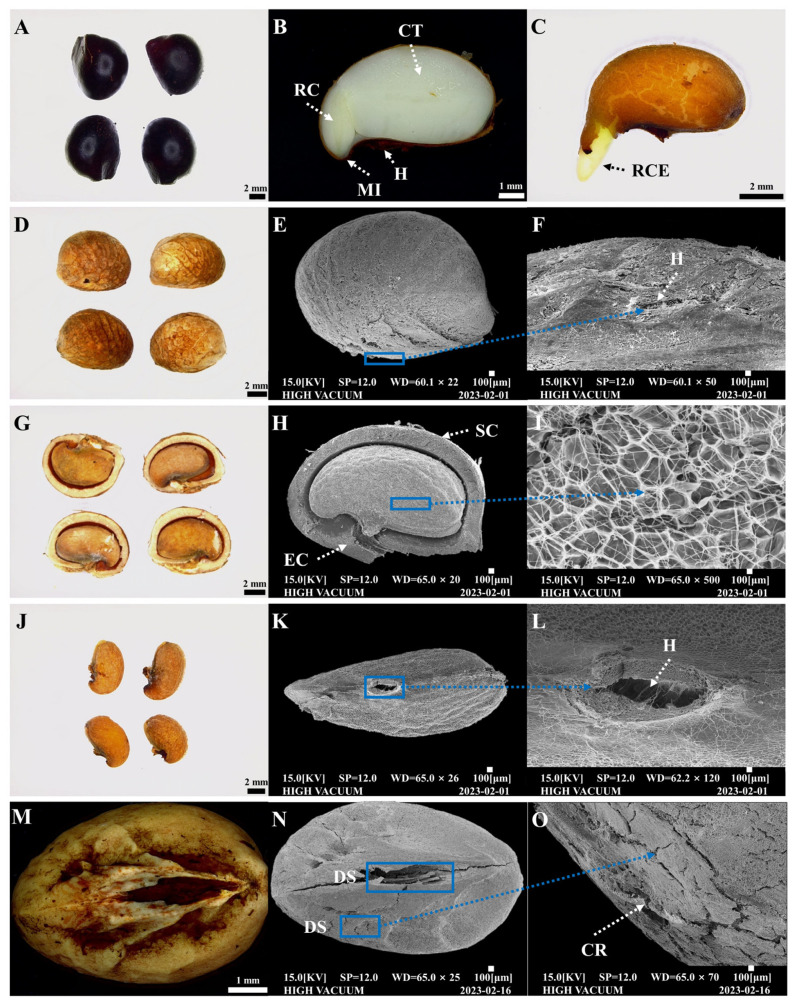
Morphology and anatomy of *Rhodotypos scandens* (Thunb.) Makino seeds. Seed with pericarp (**A**), internal view (**B**), radicle emergence (**C**), seed without pericarp (**D**–**F**), seed without endocarp (**G**–**L**), and seed scarified with sulfuric acid (**M**–**O**). CR, cracks; CT, cotyledon; DS, damaged surface; EC, endocarp; H, hilum; MI, micropyle; RC, radicle; RCE, radicle emergence; SC, seed coat.

Examinations conducted using a digital microscope and an SEM revealed that *R*. *scandens* seeds with and without pericarps displayed distinct morphoanatomical features, such as hilum, micropyle, cotyledon, radicle, and radicle emergence (Figure 2A–F). When the seed was longitudinally sectioned and the ER examined, a cross-sectional view using SEM revealed that the endocarp displayed a noticeable thickness. In addition, the internal structures of the seeds, including the hilum, were differentiated to a greater extent (Figure 2G–L). The hilum is encompassed by an endocarp distinguished by its substantial cell-wall thickness. Following the scarification of the seeds in 96% sulfuric acid for 1 h, discernible changes were observed in the external seed structure (Figure 2M–O). Specifically, pronounced fissures manifested along the major axis of the seed, accompanied by damaged surfaces and cracks in specific regions.

### 2.3. Seed Germination following Scarification, Phytohormone Treatment, and CS

**Figure 3:**
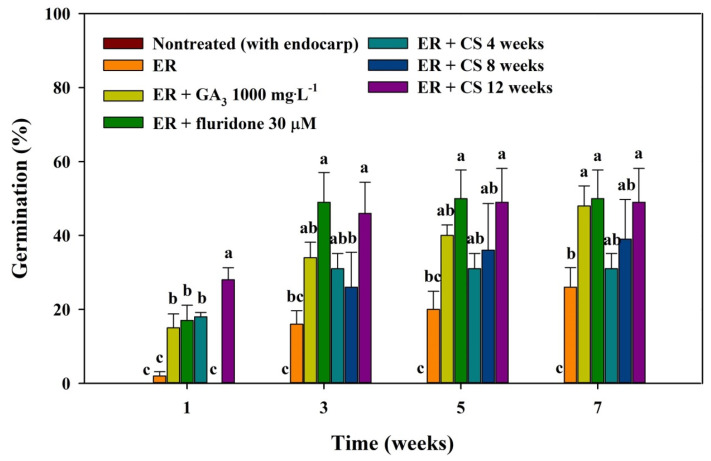
Effects of scarification, phytohormones, and cold stratification on the germination percentage of *Rhodotypos scandens* (Thunb.) Makino seeds. Vertical bars represent the standard deviation (*n* = 4). Different letters in the same column indicate significant differences based on Duncan’s multiple range test (*p* ≤ 0.05).

**Figure 4:**
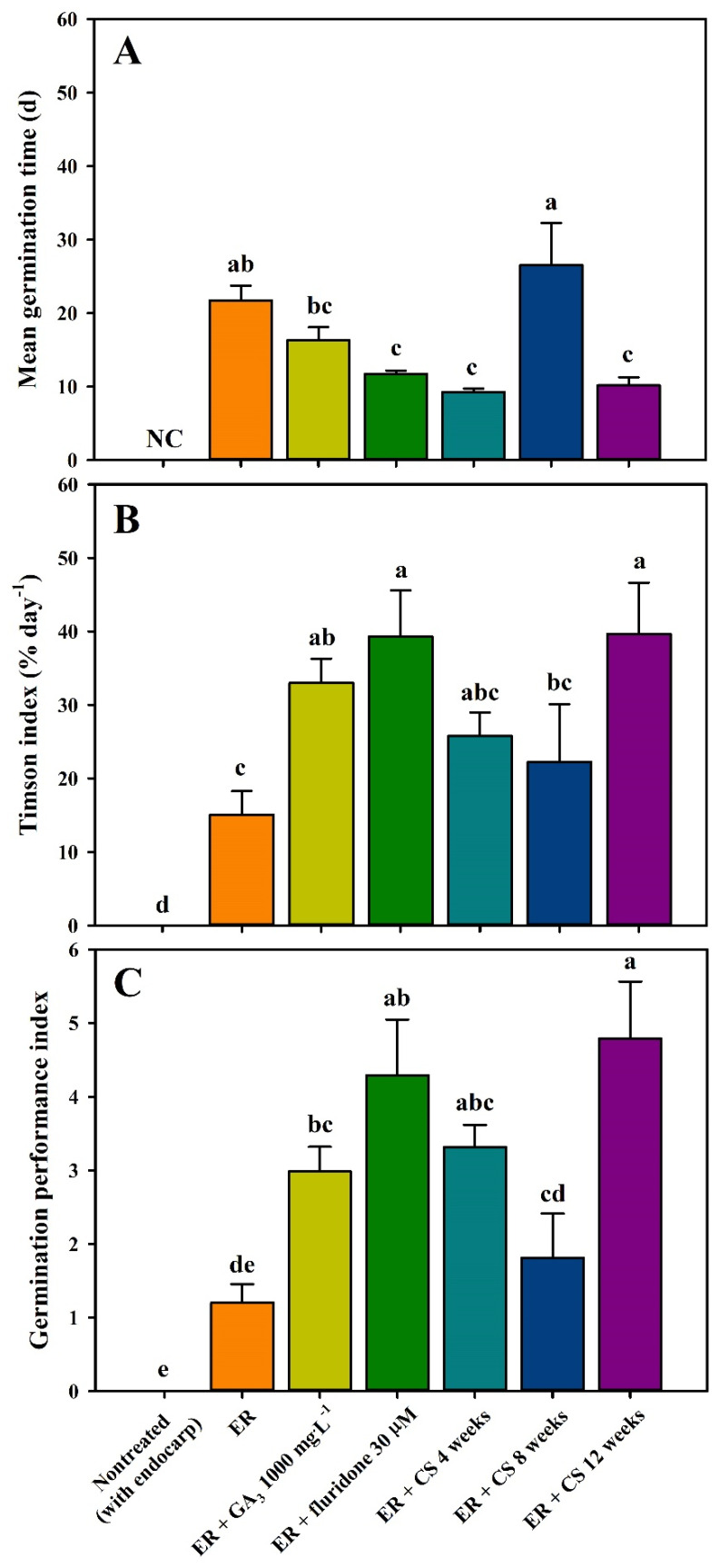
Effects of scarification, phytohormones, and cold stratification on the mean germination time (**A**), Timson index (**B**), and germination performance index (**C**) of *Rhodotypos scandens* (Thunb.) Makino seeds under seven weeks of incubation. Vertical bars represent the standard deviation (*n* = 4). Different letters in the same column indicate significant differences based on Duncan’s multiple range test (*p* ≤ 0.05). NC, not calculated.

To assess the impact of the chemical scarification of the *R*. *scandens* seeds, they were sown after immersion in sulfuric acid (for 10, 20, 30, 40, 50, and 60 min). However, none of these seeds germinated without additional treatment. On the other hand, exhibiting a stronger propensity to germinate following ER and phytohormone treatment (GA_3_ and fluridone) compared to both the nontreated (with endocarp) and ER treatment alone, the majority of seeds initiated germination within 1–3 weeks (Figure 3). Seeds treated with GA_3_ and fluridone achieved germination at a rate of 48.00 ± 5.42% and 50.00 ± 7.75% after 7 weeks, respectively. However, when exclusively subjected to ER treatment, seed germination did not exceed 26.00 ± 5.23% during the 7-week incubation period. Furthermore, nontreated seeds (with endocarps) did not exhibit germination throughout the incubation period. The application of combined treatments involving ER and CS effectively promoted root protrusion. Notably, a longer CS period positively correlated with seed germination (Figure 3). Among these treatments, a seed germination of 28.00 ± 3.27% was achieved within approximately 1 week after a CS treatment for 12 weeks. In addition, 12-week CS treatment resulted in a seed germination rate of 49.00 ± 9.15% within the 7-week incubation period. The MGT ranged from 9.2 ± 0.5 d to 26.5 ± 5.7 d. Seeds treated with a combination of ER + phytohormone and CS exhibited a decrease in MGT values compared to seeds subjected to ER alone (Figure 4). Furthermore, the combinations of ER + fluridone 30 μM and ER + CS over 12 weeks had a significant positive effect on germination characteristics, specifically TI and GPI, indicating an improvement in both the velocity and uniformity of germination.

## 3. Discussion

### 3.1. Seed Characteristics and Water Imbibition Test

The seed dormancy of PY can be identified by the occurrence of imbibition in scarified seeds, but not in intact seeds. Baskin and Baskin [16] reported that the impermeability of intact seeds, which is attributed to a thick seed coat, is a characteristic feature of PY. If the seed mass (weight) remains unchanged, this indicates that the seed (or fruit, such as endocarp + true seed) coat is impermeable to water. In contrast, if there is a greater than 20% increase in seed mass calculated from air-dried seeds or fruit mass, it can be inferred that the seed (or fruit) coat allows water permeability [33]. While nontreated *R*. *scandens* seeds (with endocarp) absorbed water, the water uptake in these species remained minimal, at approximately 27.91 ± 0.17% for 72 h (Figure 1). During seed germination, the swift uptake of water stimulates enzymatic activity, leading to seed expansion and embryo enlargement [34,35]. A comparable process of water absorption was observed in the ER-treated and water-soaked seeds of *R*. *scandens*. The ER seeds absorbed water rapidly compared to their nontreated counterparts, leading to a rapid increase in seed mass within 0–12 h of soaking. Hence, mechanically scarified ER seeds exhibited a greater capacity for water absorption compared to nontreated (with endocarp) seeds. Therefore, even though *R*. *scandens* seeds do not have PY, there may be some impediment to water absorption owing to the thick endocarp.

### 3.2. Structural Analysis of Internal and External Seed Morphoanatomy

In the present study, we observed that the seed coats of *R*. *scandens* seeds consisted of a cuticle, palisade cells, a layer of thick-walled cells, and a parenchymal cell layer (Figure 2B,H). While parenchymal cells with loose tissue exhibit a robust water absorption capacity, the cuticle, palisade cells, and thick-walled cell layers with compact structures display limited water absorption [36]. Generally, the first barrier that prevents water and oxygen from entering the seed interior is the dense cuticle layer on the surface of the seed coat, followed by the palisade layer. In this study, *R*. *scandens* seeds demonstrated a slow rate of water absorption, which was impeded by the presence of defensive cells in the thick endocarp. The cell walls of the palisade layer consist primarily of cellulose, lignin, cutin, protein, and pectin [37], which influence the thickness and hardness of the seed coat, ultimately resulting in the limited water absorption of hard seeds. However, after complete ER treatment, the hilum was clearly visible and fully exposed. This facilitated not only water absorption within the seed but also allowed seed germination and root protrusion to progress, given the removal of the thick defensive barrier. Th observation of specialized structures was not an objective of the current study. Further in-depth examination of the morphology and anatomy of *R*. *scandens* seeds, such as through dye tracking experiments, is warranted.

### 3.3. Seed Germination by Scarification, Phytohormone Treatment, and CS

Seeds of freshly ripened *R*. *scandens*, including their endocarps, were subjected to incubation under alternating temperature conditions of 25/15 °C. This resulted in a 0.00% germination rate, suggesting that the majority of seeds were dormant. Furthermore, despite the thick endocarp being damaged by the sulfuric acid treatment (Figure 2M–O), seed germination and radicle emergence did not occur. The major effect of chemical scarification on germination is the destruction of the seed coat and palisade epidermal layer, which eliminates mechanical barriers and facilitates the absorption of water or air [38]. This triggers the release of reduced amounts of sugar available for protein synthesis, ultimately promoting germination [39]. However, in the case of *R*. *scandens*, the thick endocarp poses a barrier to water absorption. In addition, the sulfuric acid treatment employed to address this issue impeded seed germination. The pericarp of rose achenes in Rosaceae is permeable and may not limit the uptake of water or oxygen by the embryo, potentially providing a mechanical barrier to embryo growth and radicle protrusion [40,41]. Furthermore, the tough endocarp was not the exclusive factor impeding germination in the studied *R*. *scandens* species, as no germination was observed when subjected solely to the sulfuric acid scarification treatment. In contrast, the mechanical scarification involving all ER seeds resulted in a radicle protrusion through the seed coat (Figure 3). In previous studies, the application of mechanical scarification was found to enhance dormancy release and promote seed germination, with similar observations being reported in numerous other plant species [15,42,43]. Therefore, similar to the mechanism of physiological germination inhibition proposed by Baskin and Baskin [17], the PD state of *R*. *scandens* seeds was primarily induced by decreased embryonic activity and the inhibitory effect of the endocarp.

In the present study, the nontreated seeds (with endocarp) showed significantly hindered germination, with only 0.00% of the seeds germinating after seven weeks of incubation. If seeds fail to germinate within 30 d under favorable germination conditions, they are deemed to be in PD [16]. According to the physiological mechanism of germination inhibition, PD can be categorized into three levels: non-deep, intermediate, and deep [17]. Recent physiological and molecular studies have demonstrated that PD comprises both embryo and coat components, with their cumulative effects and interactions dictating the extent of the overall seed PD [9]. Among the five types of dormancy, PD is the most common in temperate regions and is predominantly observed in Coniferales (Gnetales) and most angiosperm phyla [17]. Seeds can be released from PD via cold/warm stratification, after ripening, or by phytohormone treatment (GA_3_, GA_4+7_, fluridone, etc.) [12,44]. In the present study, combined the ER and phytohormone treatment had a positive effect on germination characteristics in *R*. *scandens*. GAs not only stimulate germination by counteracting the effects of ABA, but also alleviate seed dormancy [45]. The application of GAs to overcome PD has been widely utilized in studies related to seed germination and dormancy because GAs can rapidly break seed dormancy and promote germination. Further, GAs promote the synthesis of enzymes responsible for cell wall hydrolysis after seed maturation. This process accelerates radicle emergence by breaking down the endosperm barriers. Numerous studies have documented that endogenous phytohormones, particularly GAs and ABA, play a regulatory role in seed dormancy and germination [22,46,47,48]. Furthermore, the combination of fluridone, a different class of phytohormone from the GAs used in the current study, with ER exhibited positive effects on germination characteristics, as evidenced by improvements in the MGT, TI, and GPI compared to the nontreated seeds (with endocarp) (Figure 4). Fluridone, a carotenoid biosynthetic pathway inhibitor [49], promoted germination in *R*. *scandens* seeds when used in conjunction with ER (Figure 3). In previous studies, fluridone has been shown to induce seed germination in *Prunus persica*, *Caragana stipitate,* and *Nicotiana plumbaginifolia* [50,51,52]. Additionally, it is worth noting that fluridone induced an augmentation in annual *Orobanche minor* germination without diminishing endogenous ABA levels [53].

Stratification not only breaks seed dormancy through the temperature necessary for germination, but also effectively regulates germination timing [16]. Rudolf and Owston [54] reported that when *R*. *scandens* seeds were subjected to long-term stratification (approximately 120 days), seed germination increased to 80%. However, in the present study, seed germination significantly improved following a 12-week period of CS (Figure 3). Moreover, the combination of *R*. *scandens* seeds with ER and 12 weeks of CS treatment had a significant effect on germination characteristics, including MGT, TI, and GPI, ultimately leading to accelerated germination (Figure 4). In stratification experiments, the season of dispersal from the parent plant species in its native habitat under natural conditions is crucial. Seeds are typically dispersed during autumn and often overcome dormancy through the CS. Reports have indicated that seeds dispersed in autumn exhibit significant improvements in dormancy release following pretreatment with CS [20,24,44,55].

## 4. Materials and Methods

### 4.1. Seed Material

Mature seeds of *R*. *scandens* were collected on 29 September 2021 from plants growing in the Baekdudaegan National Arboretum, Bonghwa, Gyeongsangbuk-do, Republic of Korea. After the seeds were cleaned, they were examined for basic characteristics, including seed size (length × width, mm) (*n* = 10) and 1000 seed weight (g) (*n* = 100). Each parameter was measured four times, and the weight of 1000 seeds was measured using an electronic balance (ML204/01; Mettler Toledo, Columbus, OH, USA). *R*. *scandens* seeds were subjected to the drying and storage conditions (low temperature and low relative humidity (RH)) recommended by Šerá [56]. Seeds were dried in the drying room (15 °C, RH of 15%) for 2 weeks, sieved to remove plant pericarps, and then sealed in a plastic bag stored at 4 °C until use for experiments.

### 4.2. Water Imbibition Test

The permeability of seeds was determined to identify their PY under laboratory conditions (approximately 23 ± 2 °C, RH of 40–50%). Nontreated (with endocarp) and ER seeds were used in the water absorption rate test. Four replicates of 25 seeds each were initially weighed using an electronic balance. Subsequently, *R*. *scandens* seeds from each replicate were individually placed on two layers of filter paper (Whatman No. 2; Toyo Roshi Kaisha, Ltd., Tokyo, Japan) moistened with distilled water in 90 mm × 15 mm plastic Petri dishes (SPL Life Sciences Co., Ltd., Pocheon, Republic of Korea). Water was removed from the seed surfaces using paper towels, and the increase in mass for water absorption was determined after 3, 6, 9, 12, 24, 48, and 72 h of incubation. The water absorption rate of seeds was calculated using the water uptake formula [17].

W_s_ [%] = [(W_i_ − W_a_)/W_a_] × 100 (1)

where W_s_ is the increase in seed mass, W_i_ is the seed weight after a given imbibition interval, and W_a_ is the original seed weight before water absorption.

### 4.3. Structural Analysis of Internal and External Seed Morphoanatomy

The internal morphology of the seeds was examined by making cuts along their major or minor axes using a stainless-steel razor blade (Dorco, Seoul, Republic of Korea). Seeds with and without endocarp as well as seeds immersed in 96% sulfuric acid (H_2_SO_4_; Kanto Chemical Co., Inc., Tokyo, Japan) for 60 min (with endocarp) were examined for structural modifications. This analysis involved the use of intact seeds and cross-sections, which were observed using a scanning electron microscope (SEM; CX-200, COXEM, Daejeon, Republic of Korea). The surfaces and cross-sections of each treated seed were photographed using a digital microscope (DVM6; Leica Microsystems GmbH, Wetzlar, Germany) and an SEM.

### 4.4. Seed Germination following Scarification, Phytohormone Treatment, and CS

**Figure 5:**
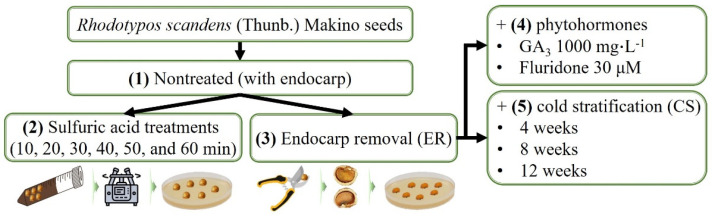
Schematic of the experimental design for the analysis of *Rhodotypos scandens* (Thunb.) Makino seed germination.

In order to determine the optimal conditions for seed propagation, the germination procedure for *R*. *scandens* seeds was conducted as follows: (1) a non-scarification treatment involving the endocarp, (2) an immersion in sulfuric acid (H_2_SO_4_, 96%) for varying durations (10, 20, 30, 40, 50, and 60 min) to assess its effect, (3) a mechanical scarification treatment without the total endocarp of seeds cut along the major axis using pruning shears, (4) an immersion in phytohormones (GA_3_ 1000 mg∙L^−1^, ≥90%, Sigma-Aldrich, St. Louis, MO, USA; and fluridone 30 μM, ≥90%, Sigma-Aldrich, St. Louis, MO, USA) for 24 h following ER, and (5) a CS (5 °C; RH of 70–90%; 4, 8, or 12 weeks) on a 1% agar medium following ER (Figure 5). After each CS, the seeds were germinated at an alternating temperature of 25/15 °C. For all seed germination experiments, the seeds were surface-sterilized by immersion in 2% NaClO for 5 min, followed by a thorough rinsing with distilled water. The seeds were induced to germinate under specific environmental conditions (at 25/15 °C, 12 h photoperiod with fluorescent lamps at 40 ± 10 μmol∙m^−2^∙s^−1^ PPFD) in a growth chamber (TGC-130H, Espec Mic Corp., Aichi, Japan) following the pre-treatment procedures. Germination experiments were performed by planting four replicates of 25 seeds each in plastic Petri dishes (90 × 15 mm; SPL Life Sciences Co., Ltd., Pocheon, Republic of Korea). These dishes were lined with 1% agar (Sigma-Aldrich, St. Louis, MO, USA) and sealed with Parafilm (PM-996, Bemis Company Inc., Neenah, WI, USA) during the incubation period.

### 4.5. Data Collection and Germination Assay

The percentage of germination (radicle protrusion from seeds) was measured every 48 h for 49 days. Each seed was considered to have germinated when the protruding radicle reached a minimum size of 2 mm. To evaluate germination, biological parameters, such as the germination (G) and mean germination time (MGT) of *R*. *scandens* seeds, were calculated as described by Ellis and Roberts [57]:

G (%) = G_49_/N × 100 (2)

MGT (days) = ∑ (T × S)/∑ S(3)

where N is the total number of seeds, T is the time in days from day one to the final day of the germination test, S is the total number of germinated seeds on day T, and G_49_ is the total number of seeds that germinated 49 days after sowing.

The germination performance index (GPI) indicates the degree of germination uniformity. *R*. *scandens* seeds were calculated using the equation described by Sundstrom et al. [58].

GPI = G/MGT(4)

A high GPI indicates a more uniform seed germination.

The rate of germination was estimated using a modified Timson index (TI) of germination velocity, as described by Khan and Gul [59]:

Timson index (% day^−1^) = ∑ (G/t) (5)

where G is the total percentage of seeds germinated every 48 h, and t is the total germination period (49 days). A high TI indicates a rapid germination rate.

### 4.6. Statistical Analysis

All data on water absorption and germination characteristics were statistically analyzed using the SAS 9.4 software (version 9.4; SAS Institute Inc., Cary, NC, USA). Significant differences in water absorption between the nontreated (with endocarp) and the ER seeds were assessed using a paired *t*-test. A one-way analysis of variance (ANOVA) was used to test the effect of factors on the G, MGT, Timson index, and GPI parameters, followed by Duncan’s honestly significant difference (HSD) post hoc test (*p* ≤ 0.05). Regression analysis and graphing were performed using SigmaPlot 12.0 (Systat Software Inc., San Jose, CA, USA).

## 5. Conclusions

Overall, *R*. *scandens* seeds exhibited a germination inhibition attributable to the rigid endocarp. However, they could not be classified as PY because water absorption proceeded without hindrance. ER treatment enhanced the germination stimulated by the application of phytohormones (GA_3_ and fluridone) and CS. Based on the aforementioned evidence, we concluded that fresh seeds of *R*. *scandens* display non-deep PD. These findings provide a valuable foundation for horticulturists and seed ecologists to further analyze the germination of *R*. *scandens* with the goal of enhancing seed propagation. Nevertheless, when considering mass propagation, manually removing the endocarp per seed is time-consuming and labor-intensive. Therefore, there is a need for a method capable of obtaining bulk ER.

## Data Availability

Data are contained within the article.
